# Role of surface tryptophan for peroxidase oxidation of nonphenolic lignin

**DOI:** 10.1186/s13068-016-0615-x

**Published:** 2016-09-17

**Authors:** Verónica Sáez-Jiménez, Jorge Rencoret, Miguel Angel Rodríguez-Carvajal, Ana Gutiérrez, Francisco Javier Ruiz-Dueñas, Angel T. Martínez

**Affiliations:** 1 CSIC, Centro de Investigaciones Biológicas, Ramiro de Maeztu 9, 28040 Madrid, Spain; 2 Department of Biology and Biological Engineering, Chalmers University of Technology, 41296 Gothenburg, Sweden; 3 CSIC, Instituto de Recursos Naturales y Agrobiología de Sevilla, Avenida Reina Mercedes 10, 41012 Seville, Spain; 4 Department of Organic Chemistry, University of Seville, Prof. García González s/n, 41012 Seville, Spain

**Keywords:** Ligninolytic peroxidases, Single-electron transfer, Catalytic tryptophan, Directed mutagenesis, Transient-state kinetics, Methylation, Acetylation, Nonphenolic lignin, Enzymatic delignification, NMR spectroscopy

## Abstract

**Background:**

Despite claims as key enzymes in enzymatic delignification, very scarce information on the reaction rates between the ligninolytic versatile peroxidase (VP) and lignin peroxidase (LiP) and the lignin polymer is available, due to methodological difficulties related to lignin heterogeneity and low solubility.

**Results:**

Two water-soluble sulfonated lignins (from *Picea abies* and *Eucalyptus grandis*) were chemically characterized and used to estimate single electron-transfer rates to the H_2_O_2_-activated *Pleurotus eryngii* VP (native enzyme and mutated variant) transient states (compounds I and II bearing two- and one-electron deficiencies, respectively). When the rate-limiting reduction of compound II was quantified by stopped-flow rapid spectrophotometry, from fourfold (softwood lignin) to over 100-fold (hardwood lignin) lower electron-transfer efficiencies (*k*_3app_ values) were observed for the W164S variant at surface Trp164, compared with the native VP. These lignosulfonates have ~20–30 % phenolic units, which could be responsible for the observed residual activity. Therefore, methylated (and acetylated) samples were used in new stopped-flow experiments, where negligible electron transfer to the W164S compound II was found. This revealed that the residual reduction of W164S compound II by native lignin was due to its phenolic moiety. Since both native lignins have a relatively similar phenolic moiety, the higher W164S activity on the softwood lignin could be due to easier access of its mono-methoxylated units for direct oxidation at the heme channel in the absence of the catalytic tryptophan. Moreover, the lower electron transfer rates from the derivatized lignosulfonates to native VP suggest that peroxidase attack starts at the phenolic lignin moiety. In agreement with the transient-state kinetic data, very low structural modification of lignin, as revealed by size-exclusion chromatography and two-dimensional nuclear magnetic resonance, was obtained during steady-state treatment (up to 24 h) of native lignosulfonates with the W164S variant compared with native VP and, more importantly, this activity disappeared when nonphenolic lignosulfonates were used.

**Conclusions:**

We demonstrate for the first time that the surface tryptophan conserved in most LiPs and VPs (Trp164 of *P. eryngii* VPL) is strictly required for oxidation of the nonphenolic moiety, which represents the major and more recalcitrant part of the lignin polymer.

**Electronic supplementary material:**

The online version of this article (doi:10.1186/s13068-016-0615-x) contains supplementary material, which is available to authorized users.

## Background

Removal of the highly recalcitrant lignin polymer is a key step for the natural recycling of plant biomass in land ecosystems, and a central issue for the industrial use of cellulosic feedstocks in the sustainable production of fuels, chemicals and different materials [1–3]. White biotechnology must contribute to the development of lignocellulose biorefineries by providing tailor-made microbial and enzymatic biocatalysts enabling “greener” and more efficient biotransformation routes for the complete use of both polysaccharides and lignin as the main biomass constituents [4, 5].

The so-called white-rot basidiomycetes (due to the whitish color of delignified wood) are the main lignin degraders in Nature [6]. The process has been described as an “enzymatic combustion” [7] and would involve peroxidases of the lignin peroxidase (LiP), manganese peroxidase (MnP) and versatile peroxidase (VP) families, together with other oxidoreductases [6, 8]. After some controversy in the past [9], the most recent evidence on the involvement of peroxidases in lignin degradation comes from the availability of massive sequencing tools applied to fungal genomes. The analysis of basidiomycete genomes shows the presence of the above ligninolytic peroxidase genes in the genomes of all typical white-rot (ligninolytic) basidiomycetes sequenced to date, and their absence from all the brown-rot (cellulolytic) basidiomycete genomes [10–14].

Among the three peroxidase families LiP, first reported from *Phanerochaete chrysosporium* [15], and VP, described later from *Pleurotus eryngii* [16, 17], have attracted the highest interest since they are able to degrade nonphenolic model compounds representing the main substructures in lignin (such as β-*O*-4′ alkyl-aryl ethers) [18–20] by single-electron abstraction forming an aromatic cation radical [21], and subsequent C_α_–C_β_ bond cleavage [22] (while MnP would act on the minor phenolic units). From the discovery of LiP, the huge number of biochemical and molecular biology studies on these enzymes generally used simple aromatic substrates, such as veratryl (3,4-dimethoxybenzyl) alcohol [23–25], and similar studies using the real lignin substrate are extremely rare [26].

A landmark in lignin biodegradation studies was the identification of a solvent-exposed peroxidase residue, Trp171 in *P. chrysosporium* LiP (isoenzyme H8) [27, 28] and Trp164 in *P. eryngii* VP (isoenzyme VPL) [29], as the responsible for oxidative degradation of nonphenolic lignin model compounds by long-range electron transfer (LRET) from the protein surface to the heme cofactor of the H_2_O_2_-activated enzyme. This single-electron transfer generates a reactive tryptophanyl radical [30, 31], whose exposed nature would enable direct oxidation of the lignin polymer. Recently, the authors have shown that removal of this aromatic residue lowers in different extents the electron transfer from technical lignins (partially phenolic softwood and hardwood water-soluble lignosulfonates) to the peroxide-activated VP transient states (the so-called compounds I and II, CI and CII) [32, 33].

To clarify the role of the surface tryptophan residue in phenolic/nonphenolic lignin degradation, stopped-flow reactions of the above VP and the corresponding tryptophan-less variant are performed in the present study using native (underivatized) and permethylated/acetylated (nonphenolic) softwood and hardwood lignosulfonates as enzyme substrates, together with lignosulfonate steady-state treatments analyzed by size-exclusion chromatography (SEC) and heteronuclear single quantum correlation (HSQC) two-dimensional nuclear magnetic resonance (2D-NMR).

## Results

### Transient kinetics of VP and its W164S variant: native lignins

Peroxidase catalytic cycle includes two-electron activation of the resting enzyme by H_2_O_2_ yielding CI, which is reduced back via CII with one-electron oxidation of two substrate molecules (Additional file 1: Figure S1a). These three enzyme forms present characteristic UV–visible spectra (Additional file 1: Figure S1b, c) that enable to calculate the kinetic constants for CI formation and CI/CII reduction (see “Methods” section).

**Fig. 1:**
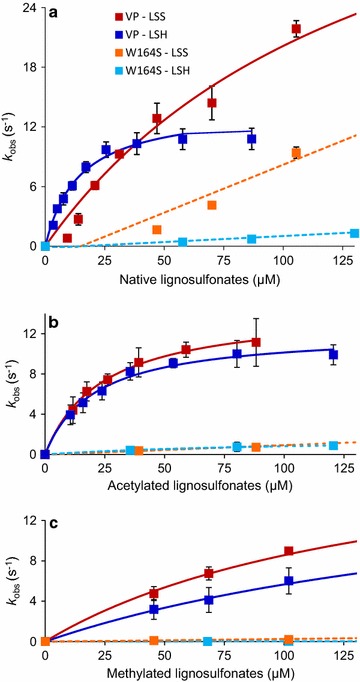
Kinetics of CII reduction by native (**a**), acetylated (**b**) and permethylated (**c**) softwood (LSS, *red*) and hardwood (LSH, *blue*) lignosulfonates: Native VP (*continuous line*) vs W164S variant (*dashed line*). Stopped-flow reactions were carried out at 25 °C in 0.1 M tartrate (pH 3). The lignosulfonate concentrations (here and in Additional file 1: Figure S2) refers to the lignosulfonate basic phenylpropanoid unit. Means and 95 % confidence limits are shown

The transient-state kinetic constants for the reaction of native lignosulfonates with H_2_O_2_-activated wild-type recombinant (hereinafter native) VP and its W164S mutated variant were obtained by stopped-flow rapid spectrophotometry, showing CII reduction as the rate-limiting step [34]. In the reactions of native VP CI and CII (Fig. 1a; Additional file 1: Figure S2a, d, continuous lines) relatively similar apparent second-order rate constants (*k*_2app_ and *k*_3app_) were obtained for the two lignosulfonates (top of Tables 1, 2) (*k*_1app_ for CI formation by H_2_O_2_ being 3460 ± 70 s^−1^ mM^−1^). The main difference was in the CII reduction dissociation constant (*K*_D3_), which was tenfold lower for hardwood than softwood lignosulfonate indicating a higher affinity for the former lignin. Softwood lignosulfonate did not saturate native VP for CI reduction (Additional file 1: Figure S2a, d, red continuous line) and only a *k*_app_ value can be provided.

**Table 1:** CI reduction constants by acetylated, methylated and native (softwood and hardwood) lignosulfonates: Native VP vs W164S variant

	Softwood lignin			Hardwood lignin		
	*Acetylated*	*Methylated*	*Native*	*Acetylated*	*Methylated*	*Native*
*Native VP*						
*k*_2_ (s^−1^)	35.7 ± 3.5	–	–	25.9 ± 1.9	8.4 ± 0.9	188 ± 7
*K*_D2_ (μM)	111 ± 19	–	–	91 ± 13	66 ± 17	38 ± 3
*k*_2app_ (s^−1^ mM^−1^)	320 ± 24	101 ± 8	2080 ± 80	289 ± 22	128 ± 19	4950 ± 190
*W164S variant*						
*k*_2_ (s^−1^)	–	8.9 ± 2.7	–	7.9 ± 0.4	–	–
*K*_D2_ (μM)	–	355 ± 178	–	122 ± 11	–	–
*k*_2app_ (s^−1^ mM^−1^)	60 ± 3	25 ± 5	627 ± 87	65 ± 2	12 ± 2	540 ± 15

**Table 2:** CII reduction constants by acetylated, methylated and native (softwood and hardwood) lignosulfonates: Native VP vs W164S variant

	Softwood lignin			Hardwood lignin		
	*Acetylated*	*Methylated*	*Native*	*Acetylated*	*Methylated*	*Native*
*Native VP*						
*k*_3_ (s^−1^)	14.4 ± 0.4	21.2 ± 2.0	48 ± 2	12.2 ± 0.5	18.4 ± 1.6	14 ± 1
*K*_D3_ (μM)	24.1 ± 1.9	147 ± 25	143 ± 19	20.6 ± 2.5	226 ± 33	14 ± 2
*k*_3app_ (s^−1^ mM^−1^)	599 ± 31	144 ± 10	340 ± 30	592 ± 52	82 ± 5	990 ± 80
*W164S variant*						
*k*_3_ (s^−1^)	–	–	–	1.6 ± 0.2	–	–
*K*_D3_ (μM)	–	–	–	98.2 ± 22.5	–	–
*k*_3app_ (s^−1^ mM^−1^)	9.0 ± 0.8	3.0 ± 0.3	96 ± 12	16 ± 2	0.23 ± 0.07	8 ± 0.1

In the W164S variant (whose no-saturation kinetic traces are included in Fig. 1a; Additional file 1: Figure S2a, d, dashed lines) substitution of the catalytic tryptophan resulted in impaired oxidation of both lignosulfonates (bottom of Tables 1, 2). The strongest effect was with the hardwood lignosulfonate, where the *k*_2app_ and rate-limiting *k*_3app_ values experienced ninefold and 125-fold decreases, respectively.

### Transient kinetics of VP and its W164S variant: nonphenolic lignins

**Fig. 2:**
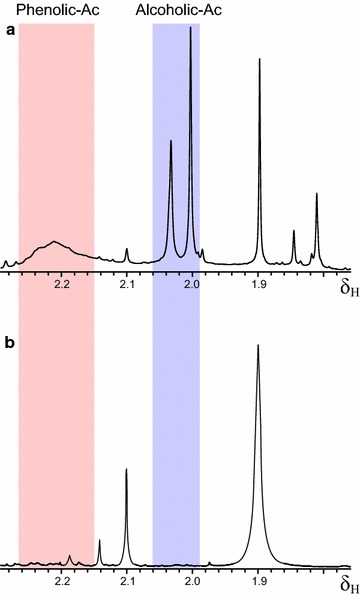
Lignosulfonate permethylation: ^1^H-NMR analysis after secondary acetylation confirming the previous complete methylation of softwood lignosulfonate (**b**) compared with the untreated sample (**a**). Regions of phenolic and alcoholic acetates are indicated

The residual reduction of W164S CI and CII in the above experiments could be due to the presence of more easily oxidizable phenolic units. Using NMR after sample acetylation, the lignosulfonate phenolic content was estimated as ~20–30 % of lignin units. Methylation was optimized using pyrolysis–gas chromatography/mass spectrometry (Py-GC/MS) to follow the reaction progress (Additional file 1: Figure S3) till complete derivatization (of both phenolic and alcoholic hydroxyls), as shown by NMR after secondary acetylation (Fig. 2).

Then, new transient-state kinetic constants were calculated for the derivatized (nonphenolic) lignosulfonates. Figure 1b, c (and Additional file 1: Figure S2b/e, c/f) show the kinetic traces for the acetylated and methylated lignosulfonates, respectively, whose CI and CII reduction constants are included in Tables 1 and 2, respectively. With these nonphenolic lignins no strong difference between CI and CII reduction rates was observed, in contrast with native lignosulfonate where CII reduction is clearly the rate-limiting step. In most native VP reactions (continuous lines), saturation kinetics was observed (except for CI reduction by methylated softwood lignosulfonate) and only a *k*_2app_ value can be provided. The opposite tendency was found for the W164S variant (dashed line) where saturation was more rarely observed.

For native VP, lignin methylation (and in lower extent acetylation) significantly decreased CI reduction (Additional file 1: Figure S2, left) resulting in 20–40-fold lower *k*_2app_ values, while CII reduction was much less affected (Fig. 1). However, for the W164S variant, similar decreases in both CI and CII reduction were observed, resulting in 25–45-fold lower *k*_app_ for the methylated samples.

When the effect of W164S mutation on the nonphenolic lignin constants was considered (bottom of Tables 1, 2), small decreases in CI reduction were observed (similar to those obtained with native lignins). However, for reduction of W164S CII strong *k*_3app_ decreases with respect to native VP were observed (up to 350-fold for the methylated hardwood lignosulfonate). More importantly, the previously observed reduction of W164S CII by native softwood lignosulfonate (Fig. 1a, red dashed line) disappeared when the acetylated or methylated samples were evaluated as W164S substrates (Fig. 1b, c red dashed lines, respectively).

### Steady-state treatment of native lignin with VP and its W164S variant

In addition to the above stopped-flow reactions, the effect of the enzymatic treatments was also analyzed by SEC and 2D-NMR spectroscopy during steady-state reactions.

**Fig. 3:**
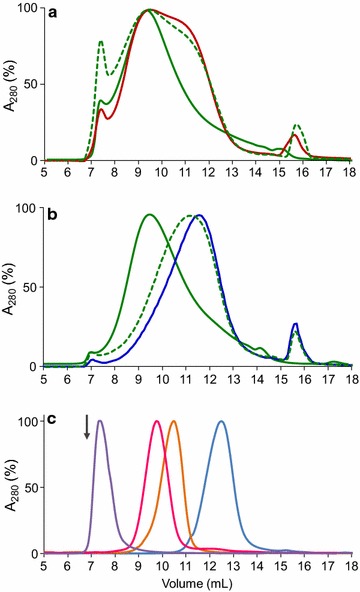
SEC profiles of softwood (**a**) and hardwood (**b**) lignosulfonates treated for 24 h with native VP and its W164S variant and control without enzyme, and sulfonated polystyrene standards (**c**). Lignosulfonate samples (12 g L^−1^) after a 24-h treatment with 1.2 µM native VP (*green line*) and its W164S variant (*dashes*) in presence of 9.5 mM H_2_O_2_, and the corresponding softwood (*red*) and hardwood (*blue*) lignosulfonate controls without enzyme, were analyzed in a Superdex-75 column using 0.15 M NaOH as eluent (0.5 mL·min^−1^) and detection at 280 nm. Sulfonated polystyrenes (Mp 78,400, 29,500, 10,200 and 4210 Da, from left to right) were used as molecular mass standards in **c** (arrow shows the excluded blue dextran elution volume)

Native VP significantly modified the molecular-mass distribution and main peak (Mp) of softwood and especially of hardwood lignosulfonates (green continuous lines in Fig. 3a, b, respectively), with respect to the controls (red and blue lines), revealing a clear polymerization tendency in the latter case (Mp of ~20,000 Da compared with ~5500 Da in the control) and the disappearance of a broad shoulder around 11 mL elution volume (~6800 Da) in the former case. More importantly, the W164S variant only caused a very limited modification in the molecular-mass distribution of the two lignins, in agreement with its low kinetic constants for rate-limiting CII reduction. Such modification included a modest displacement of Mp (to 6500 Da) in hardwood lignosulfonate and a slight decrease of the softwood lignosulfonate shoulder (dashed lines).

**Fig. 4:**
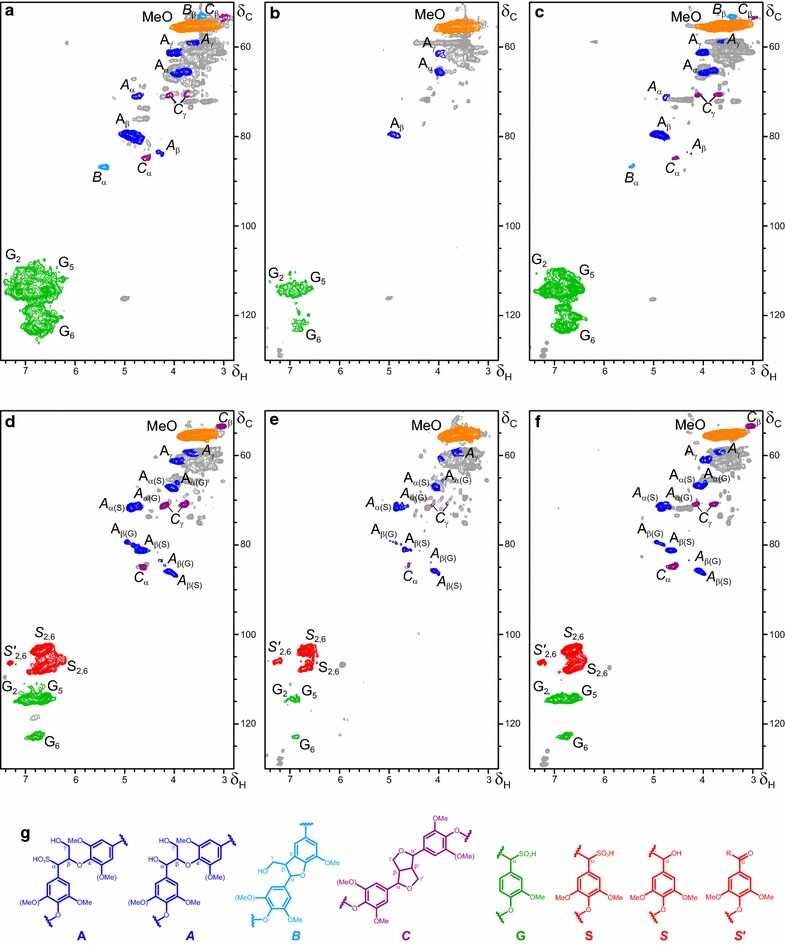
HSQC NMR spectra of softwood (**a–c**) and hardwood (**d–f**) lignosulfonates treated for 24 h with native VP (**b**, **e**) and its W164S variant (**c**, **f**) and control without enzyme (**a**, **d**), and formulae of the main structures identified (**g**). Signals correspond to ^13^C-^1^H correlations at the different positions of lignin native/α-oxidized/α-sulfonated syringyl (*red signals*) and guaiacyl (*green signals*) units, α-sulfonated/non-sulfonated side chains in β-*O*-4′ (*blue signals*), phenylcoumaran (*cyan signals*), and resinol (*purple signals*) substructures, and methoxyls (*orange signal*) (*gray*, unassigned signals). Same amount of sample (40 mg before enzymatic treatment) and DMSO-*d*_*6*_ (0.75 mL) were used for all the spectra, which were normalized to the same intensity of the DMSO signal (not shown) for comparison. *List of signals* (δ_C_/δ_H_ ppm): 53.2/3.46, C_β_/H_β_ in phenylcoumarans (***B***_**β**_); 53.4/3.00, C_β_/H_β_ in resinols (***C***_**β**_); 55.5/3.66, C/H in methoxyls (**MeO**); 59.4/3.4 and 3.72, C_γ_/H_γ_ in β–*O*–4′ (***A***_**γ**_); 61.1/4.00, C_γ_/H_γ_ in sulfonated β–*O*–4´ (**A**_**γ**_); 65.6/3.93, C_α_/H_α_ in sulfonated β–*O*–4′ linked to a G-unit (**A**_**α(G)**_); 67.2/4.02, C_α_/H_α_ in sulfonated β–*O*–4′ linked to a S-unit (**A**_**α(S)**_); 70.8/4.16 and 3.77, C_γ_/H_γ_ in β-β′ resinols (***C***_**γ**_); 71.1/4.72, C_α_/H_α_ in β–*O*–4′ linked to a G-unit (***A***_**α(G)**_); 71.5/4.85, C_α_/H_α_ in β–*O*–4′ linked to a S-unit (***A***_**α(S)**_); 79.3/4.91, C_β_/H_β_ in sulfonated β–*O*–4′ linked to a G unit (**A**_**β(G)**_); 80.9/4.67, C_β_/H_β_ in sulfonated β–*O*–4′ linked to a S unit (**A**_**β(S)**_); 83.3/4.24, C_β_/H_β_ in β–*O*–4′ linked to a G unit (***A***_**β(G)**_); 84.9/4.59, C_α_/H_α_ in β-β′ resinols (***C***_**α**_); 85.7/4.08, C_β_/H_β_ in β–*O*–4′ linked to a S unit (***A***_**β(S)**_); 86.7/5.41, C_α_/H_α_ in phenylcoumarans (***B***_**α**_); 103.8/6.68, C_2_/H_2_ and C_6_/H_6_ in syringyl units (***S***_**2,6**_); 106.2/7.29, C_2_/H_2_ and C_6_/H_6_ in α-oxidized syringyl units (***S’***_**2,6**_); 108.0/6.68, C_2_/H_2_ and C_6_/H_6_ in sulfonated syringyl units (**S**_**2,6**_); 114.0/6.60 and 114.3/6.87, C_2_/H_2_ and C_5_/H_5_ in guaiacyl units (**G**_**2**_ **/G**_**5**_); and 122.8/6.75, C_6_/H_6_ in guaiacyl units (**G**_**6**_) (minor, and largely overlapping, signals of C_2_/H_2_, C_5_/H_5_ and C_6_/H_6_ correlations in non-sulfonated guaiacyl units would appear at 110.7/6.93, 114.2/6.65 and 118.6/6.79 ppm, respectively; not shown). Three additional aromatic signals in the treated samples, at 126.1/7.14, 127.7/7.21 and 128.9/7.22 ppm, were assigned to protein (phenylalanine residues in the added enzyme)

Then, the structural modifications of guaiacyl (**G**) and syringyl-guaiacyl (**S–G**) lignins from softwood and hardwood, respectively, were analyzed by 2D-NMR (Fig. 4). The main lignosulfonate units and side-chain interunit linkages are shown in Fig. 4g (no *p*-hydroxyphenyl units were detected). Both sulfonated (**A**) and non-sulfonated (***A***) β-*O*-4′ substructures were found in the control lignins, together with less abundant (non-sulfonated) phenylcoumaran (***B***) and resinol (***C***) substructures (Fig. 4a, d).

After 24-h treatment with native VP (Fig. 4b, e) the intensity of the signals of the different aromatic lignin units (**G**, **S,** ***S*** and ***S*** **′**) and side-chain interunit linkages (**A**, ***A***, ***B*** and ***C***) decreased simultaneously, maintaining similar linkage percentages. However, the methoxyl numbers per unit increased up to ~ twofold. In the hardwood lignosulfonate, this was accompanied by higher abundance of C_α_-oxidized syringyl units (***S′***) with respect to total syringyl units, while the S/G ratio also increased (from 2.0 in the control to 3.5 in the 24-h treated sample). Concerning side-chain signals, only those of the main sulfonated β-*O*-4′ substructures (**A**_**α**_, **A**_**β**_ and **A**_**γ**_) remained in the softwood lignosulfonate, while those of phenylcoumaran (***B***), resinol (***C***) and β-*O*-4′ (***A***) non-sulfonated side chains disappeared. In contrast, signals of sulfonated (**A**) and non-sulfonated β-*O*-4′ (***A***) and resinol (***C***) side chains could be observed in the hardwood lignosulfonate, albeit with low intensities. More interestingly, in the lignosulfonates treated for 24 h with the W164S variant (Fig. 4c, f) only minor changes in the aliphatic/aromatic HSQC signals were observed (spectra with similar intensities of most signals, and only slight increases of methoxyl content and S/G ratio compared with the control).

### Steady-state treatment of nonphenolic vs native lignosulfonates

With the purpose of further investigating lignosulfonate modification by VP, including the observed small changes by the W164S variant, derivatized (nonphenolic) lignosulfonates were treated in new steady-state experiments.

The native VP was able to modify the nonphenolic lignosulfonates but the changes in the molecular-mass distribution (Additional file 1: Figure S4, green continuous line) and molecular structure of lignins (Additional file 1: Figure S5b, e) were modest, compared with those observed for the native (partially phenolic) lignosulfonates (Fig. 3a, b, green continuous line, and Fig. 4b, e, respectively). These modifications include lower-intensity signals in the NMR spectra of nonphenolic hardwood lignosulfonate (the ***S′*** signal being the exception) and displacement of the Mp in the SEC profile, while lower changes were observed for the nonphenolic softwood lignosulfonate.

In contrast, the SEC profiles of the W164S-treated (green dashed lines) and control (red and blue lines) lignosulfonates were very similar (Additional file 1: Figure S4), and the same was observed in the 2D-NMR spectra (Additional file 1: Figure S5 c/f, a/d, respectively). The above results indicate that the phenolic lignin moiety: (i) promoted lignosulfonate modification under steady-state conditions; and (ii) was also responsible for the (small) modification of native lignin by the W164S variant.

### Comparison with *P. chrysosporium* LiP

The transient state kinetic constants for reduction of *P. chrysosporium* LiP CII (the rate-limiting step in catalytic cycle) by native and methylated (nonphenolic) softwood and hardwood lignosulfonates were obtained (Additional file 1: Figure S6). Only the hardwood lignosulfonate (blue continuous line) showed saturation kinetics, with *k*_3_ and *K*_D3_ values in the same order of those obtained for VP. In contrast, the *k*_3app_ value for softwood lignosulfonate was over one order of magnitude lower than found for VP. Methylation reduced the electron transfer rate (*k*_3app_) and for the hardwood lignosulfonate this decrease was much higher than found for VP.

SEC and 2D-NMR spectroscopy of lignosulfonates treated with LiP revealed the same modification trends of the VP treatments. The shoulder (~11 mL) in softwood lignosulfonate SEC (Additional file 1: Figure S7a, red) was reduced without Mp displacement (green line), while in hardwood lignosulfonate (Additional file 1: Figure S7b) the Mp was displaced (~7500 Da) but the polymerization degree was lower than found with VP. Most signals in the 2D-NMR spectra of softwood (Additional file 1: Figure S8a–c) and hardwood (Additional file 1: Figure S8d–f) lignosulfonates showed slightly reduced intensities after LiP treatment, although the decreases were much lower than found with VP. The only exception was the (C_α_-oxidized) *S*′ units increasing after the LiP treatment, as also found for VP. Moreover, the main changes were produced during the first 3 h, while with VP they progressed during the whole treatment.

## Discussion

### Etherified lignin from phenolic monolignols

Although lignin precursors (monolignols) are phenolic (4-hydroxycinnamylic) alcohols, the resulting polymer is basically nonphenolic, since β-*O*-4′ and other ether interunit linkages involving the C_4_ hydroxyl (as found also in phenylcoumaran and 4-*O*-5′ structures) are predominant [35, 36]. In this way, only average 15 % phenylpropanoid units in native (natural) lignins would be phenolic [37], with the highest contents in grasses and conifers [38, 39]. This phenolic content increases in technical (industrial) lignins since cleavage of some interunit ether linkages is always produced in chemical pulping [40]. However, while kraft pulping (the most widespread process nowadays) is largely based on lignin (ether linkage) breakdown releasing a highly phenolic (up to 70 % or more) lignin, the sulfite process is based on lignin solubilization (after sulfonation) resulting in only moderate increases of the phenolic content [38, 41]. These two lignosulfonate characteristics (water solubility and phenolic content more similar to native lignin) were exploited in the present study to estimate electron transfer rates between (native and derivatized) lignin and ligninolytic peroxidases (including a mutated VP variant).

### Electron transfer as seen from the peroxidase side (stopped-flow data)

Accurate kinetic constants for lignin degradation (by basidiomycete peroxidases) are difficult to be obtained under steady-state conditions due to the impossibility to follow lignin oxidation during short incubation periods (for maximal enzyme activity). However, the corresponding electron transfer rates can be precisely estimated (from the “peroxidase side”) by following the reduction of the H_2_O_2_-activated enzyme transient states (CI and CII) by lignin, using rapid spectrophotometry in single-turnover reactions under stopped-flow conditions.

Transient-state kinetic constants for *P. chrysosporium* LiP reduction by in vitro synthesized lignin (dehydrogenation polymer, DHP) had been reported (with *k*_3app_ ~60 s^−1^ mM^−1^) [26], the differences with the LiP constants obtained here being most probably related to differences in lignin preparations and solubility limitations using DHP. Also, DHP has a significant phenolic content [42] that will affect electron-transfer estimation, as shown here for lignosulfonates. Moreover, no mutated variants were included in these LiP studies [26] and, therefore, the catalytic residue/s remained unidentified. The first evaluation of several (three) possible LRET pathways for peroxidase oxidation of lignin was reported for *P. eryngii* VP [29] showing that only the pathway initiated at Trp164, homologous to LiP Trp171 [27], was operative. The VP and LiP site-directed mutagenesis studies used VA as a simple model for nonphenolic lignin. Other nonphenolic compounds (from dimers to tetramers) including the lignin most frequent linkages were used in subsequent studies [18, 20, 28, 43] but site-directed mutagenesis studies using the lignin polymer as substrate have been only recently reported, as discussed below.

Using water-soluble lignosulfonates, we estimated the reduction constants of *P. eryngii* VP transient states and, unexpectedly, some reduction of both CI and CII was observed for the W164S variant lacking the putative catalytic residue [32]. In the present study, we compared the transient-state kinetic constants of *P. eryngii* VP (and its W164S variant) and *P. chrysosporium* LiP on native (20–30 % phenolic) and nonphenolic (derivatized) softwood and hardwood lignosulfonates. With this purpose, samples were methylated with methyl iodide [44], which has advantages with respect to other methylating agents applied to lignosulfonates [45, 46].

First, we found that lignin methylation and acetylation—introducing ether (as found in nonphenolic lignin) and ester linkages at the phenolic hydroxyls, respectively—significantly lower the electron transfer rates, indicating that the phenolic units are easier to be oxidized by the enzyme. The above correlated with the lower lignin modification after steady-state treatment discussed below. Preferential degradation of the phenolic lignin moiety had been described after fungal decay by *P. eryngii* [47]. In spite of the above decrease of electron transfer rates, the constants for VP CI and CII reduction by the nonphenolic lignosulfonates (*k*_2app_ 100–320 and *k*_3app_ 80–600 s^−1^ mM^−1^) are much higher than reported for veratryl alcohol (*k*_2app_ 2.8 and *k*_3app_ 1.3 s^−1^ mM^−1^) [48]. This is mainly due to lower *K*_D_ revealing that VP is more efficient binding polymeric lignin than simple aromatics. Moreover, although LiP is better reduced by veratryl alcohol [49, 50] than VP, its reduction constants by nonphenolic lignosulfonates are worst that found for VP, indicating that VP is more efficient than LiP abstracting electrons from nonphenolic lignin (under the present experimental conditions). This correlates with the significantly higher lignosulfonate modification found after VP treatment.

Second, and more importantly, we demonstrated that the solvent-exposed catalytic tryptophan (Trp164 of *P. eryngii* VP) is required for oxidizing the main nonphenolic lignin moiety, since CII reduction is practically absent in the W164S mutated variant. This is shown by both transient-state kinetic constants (50–60 fold lower *k*_3app_ values for nonphenolic than native lignin) and SEC and 2D-NMR results. Since they have a similar phenolic moiety, differences between CII reduction by the two native lignosulfonates could be related to the smaller size of the monomethoxylated units in softwood lignin, enabling contact and direct electron transfer to the heme cofactor at the main access channel. In contrast, we found that nonphenolic lignin can reduce the CI of the W164S variant, although with only 20–25 % efficiency compared with native VP. The above suggests that in native VP catalytic cycle (Additional file 1: Figure S1a) the Trp164 radical is required for nonphenolic lignin oxidation at the CII level (VP-II_B_) while at the CI level both the porphyrin radical (VP-I_A_) and the Trp164 radical (VP-I_B_) would be able to oxidize nonphenolic lignin.

### Additional aspects of lignin modification as shown by SEC and 2D-NMR

2D-NMR spectroscopy represents the state-of-the-art technology for structural characterization of lignins [51–53], with broad application to lignin-engineered transgenic plants for biorefineries [54, 55]. This technique has been also used to study delignification of lignocellulosic feedstocks by fungal laccases in the presence of redox mediators [56, 57]. In a recent study, the authors used for the first time 2D-NMR to demonstrate lignosulfonate degradation by VP [32, 33].

After assigning the main signals of sulfonated and non-sulfonated lignin structures, their 2D-NMR spectra (normalized to the same amount of sample at the beginning of treatment and the same solution volume in the NMR tubes) showed (i) from small to large decreases in the intensity of the above signals and (ii) variable structural modifications of lignins, during their steady-state treatment (the extent of the above changes is clearly illustrated in the difference spectra of softwood and hardwood lignosulfonates—treated samples minus their controls—included as Additional file 1: Figure S9, S10, respectively). In laccase-mediator treatment of lignosulfonates, the decrease of HSQC signals was mainly due to the condensation reactions giving rise to quaternary (unprotonated) carbons [58]. However, degradation of lignin aromatic (and aliphatic) structures is produced during VP treatment, as shown by ^13^C NMR spectroscopy [32]. Unexpectedly, VP caused a stronger modification than LiP, resulting in the disappearance (or strong decline) of lignin signals. The observed increase of methoxyls (per aromatic unit) suggests the formation of non-aromatic methoxyl-containing (e.g. muconate type) structures [59]. The relative abundance of (C_α_-oxidized) *S*′ units also increased in the treated lignins, as previously reported for the lignin-degrading laccase-mediator system [57, 60]. Such oxidation is among the first reactions in lignin biodegradation.

In contrast with the above results using native (unmodified) peroxidase, the VP variant lacking surface Trp164 only caused a modest modification of the NMR spectra, confirming that its lignin-degrading ability is largely associated to the presence of this surface residue. Moreover, when derivatized lignosulfonates were treated with the Trp164-less variant, the spectra were superimposable to those of the enzyme-less controls, demonstrating that this catalytic residue is strictly required for degradation of the nonphenolic lignin.

In addition to the structural modification revealed by 2D-NMR, the SEC profiles revealed repolymerization of a part of the products from lignin degradation by VP, resulting in residual lignins with increased molecular masses. This behavior, which is due to the coupling tendency of phenoxy and other aromatic radicals already reported in early “ligninase” studies [61], has been described for other oxidoreductases [62–64], being especially remarkable in laccase-mediator treatments [58].

## Conclusions

Data from stopped-flow (single turnover) analyses and steady-state treatments (the latter analyzed by SEC and 2D-NMR) of native and derivatized (nonphenolic) lignosulfonates unambiguously demonstrate that: (i) the minor phenolic moiety of lignin is preferentially degraded by ligninolytic VP; and (ii) a solvent exposed tryptophan residue (conserved in both VPs and LiPs) is required for electron transfer between the nonphenolic lignin and the H_2_O_2_ activated enzyme.

## Methods

### Enzyme production

Native VP from *P. eryngii* (mature protein-coding sequence of isoenzyme VPL2, GenBank AF007222) and its W164S mutated variant [29] were produced in *Escherichia coli* and in vitro activated as reported elsewhere [65]. The mature protein-coding sequence of *P. chrysosporium* LiP-H8 (GenBank Y00262) was also produced in *E. coli* and in vitro activated [66, 67].

The recombinant enzymes were purified by anion-exchange chromatography (Resource Q column, GE Healthcare, Uppsala, Sweden) using a 0–0.3 M NaCl gradient (2 mL min^−1^, 20 min) in 1 mM CaCl_2_-containing 10 mM tartrate, pH 5.5 (for VP and its W164S variant), or succinate, pH 6 (for LiP). The *R*_z_ (A_410_/A_280_ ~4) values were indicative of the purity of the enzymes, and the electron absorption spectra confirmed the correct folding and cofactor incorporation.

### Native and derivatized softwood and hardwood lignins

Two water-soluble sulfonated lignins were used in this study: softwood (*Picea abies*) and hardwood (*Eucalyptus grandis*) lignosulfonates kindly provided by G. E. Fredheim (Borregaard AS, Sapsborg, Norway). The lignosulfonate samples were dialyzed in 10 mM EDTA, 50 mM Tris (pH 8) with the aim of removing Mn^2+^ traces (which reduce H_2_O_2_-activated VP), and then in Milli-Q water.

Lignosulfonates (50 mg) were acetylated in a 50-mL pear-shaped flask with 3 mL of a pyridine-acetic anhydride (1:1, v/v) solution, stirring for 24 h at room temperature. Then, 10 mL of aqueous methanol (50 %) were added and the mixture was evaporated to dryness under vacuum. The solvent treatment was repeated three times with toluene (3 × 10 mL), and once with methanol (10 mL). Finally, the acetylated lignosulfonates (60–65 mg) were dried at 50 °C overnight. Acetylated lignosulfonates were used as enzyme substrate, and for estimation of phenolic and alcoholic hydroxyl content by NMR, as described below.

For lignosulfonates *O*-methylation with methyl iodide [44, 68], ~65 mg of sample were dissolved in 10 mL of dimethylsulfoxide (DMSO), methyl iodide (1 mL) and finely powdered NaOH (1 g) were added, and the mixture was vigorously vortexed for 10 min. Then, additional NaOH (300 mg) and methyl iodide (1 mL) were added, the mixture was stirred for 1 h, and the reaction quenched by adding 10 mL of water and adjusting the pH below 7 with 1 M HCl. The methylated lignosulfonates (45–55 mg) were dialyzed, concentrated under vacuum and freeze-dried.

### Enzyme (transient-state) kinetics

Reduction of peroxidase CI and CII in 0.1 M tartrate (pH 3) by softwood and hardwood lignosulfonates (native and derivatized samples) was followed in a stopped-flow rapid spectrophotometry equipment (Bio-Logic, Claix, France) with a three-syringe module (SFM300) synchronized to a diode array detector (J&M, Essingen, Germany), and BioKine software.

CI reduction was studied by mixing the enzyme (1 µM final concentration) with H_2_O_2_ (1 µM final concentration) for 0.6 s, resulting in CI formation. Next, different amounts of lignosulfonate (5–350 µM final concentration) in 0.1 M (final concentration) tartrate (pH 3) were added, and the reactions were followed at 416 nm (isosbestic point of VP CII and resting state). CII reduction was studied by mixing a solution of enzyme and ferrocyanide (both at 1 µM final concentration) with H_2_O_2_ at equimolar ratio. The solution was aged for 6 s, and CII formation was achieved. Then, different amounts of lignosulfonate (5–350 µM final concentration) in 0.1 M (final concentration) tartrate (pH 3) were added, and the reaction was followed at 406 nm (Soret maximum of resting VP and LiP). The lignin concentrations in these and other experiments were referred to the basic phenylpropanoid unit in softwood and hardwood lignosulfonates.

All kinetic traces exhibited single-exponential character from which pseudo first-order rate constants (*k*_2obs_ and *k*_3obs_ for CI and CII reduction, respectively) were calculated. Plots of *k*_2obs_ and *k*_3obs_ vs substrate concentration fitted to linear or hyperbolic models. From those kinetics that fitted to a linear model apparent second-order rate constants (*k*_2app_ and *k*_3app_ for CI and CII reduction, respectively) were obtained. Plots of *k*_obs_ vs substrate concentration that fitted to a Michaelis–Menten model yielded dissociation constants of the CI-lignin and CII-lignin complexes (*K*_D2_ and *K*_D3_, respectively) and first-order rate constants (*k*_2_ and *k*_3_, respectively). The corresponding apparent second-order rate constants, *k*_2app_ (*k*_2_/*K*_D2_) and *k*_3app_ (*k*_3_/*K*_D3_), were calculated with the equation: *k*_obs_ = (*k*/*K*_D_)[S]/(1 + [S]/*K*_D_), where [S] indicates substrate concentration.

### Lignin treatment under steady-state conditions

Lignosulfonates (12 g L^−1^) were treated with VP, its W164S variant, and LiP (all 1.2 µM concentration, added in two doses at the beginning and after 6 h of reaction) and H_2_O_2_ (9.5 mM, final concentration, added continuously over 24 h with a syringe pump) in 50 mM phosphate (pH 5), at 25 °C, and samples were taken after different times (3, 12 and 24 h). Control treatments were performed under the same conditions but in the absence of enzyme. Although VP and LiP show the highest activity at pH 3 (as used in stopped-flow experiments) the above long-term lignosulfonate treatments were performed at pH 5 (to maintain the enzyme active during the whole incubation period) after preliminary experiments where treatments at pH 3.5 and 5 were compared.

### SEC analyses

Changes in the molecular-mass distribution of lignosulfonates after 24-h peroxidase treatment and controls were analyzed by SEC using a Superdex-75 column (HR-10/30, 3000–70,000/100,000 Da range; GE Healthcare) with 0.15 M NaOH as the mobile phase, at a flow rate of 0.5 mL·min^−1^, and UV (280 nm) detection. Blue dextran (Serva, Heindelberg, Germany) was used to determine the exclusion volume of the column, and a kit of sulfonated polystyrenes sodium salt standards with Mp in the 4210–976,000 Da range (PSS, Mainz, Germany) was used for calibration and mass determination (Ve/Vo vs Log[Mp], where Ve and Vo are the elution and void volumes respectively).

### NMR analyses

Samples after different times (3, 12 and 24 h) of native and derivatized lignosulfonate treatment and the corresponding controls were freeze-dried for NMR analyses. Solution NMR spectra, including ^1^H-NMR and HSQC 2D-NMR, were recorded at 25 °C on an AVANCE III 500 MHz instrument (Bruker) equipped with a cryogenically cooled 5 mm TCI gradient probe with inverse geometry. The lignosulfonate samples (40 mg initial weight, before treatments) were dissolved in 0.75 mL of deuterated DMSO-*d*_6_. The central solvent peak was used as the internal reference (at δ_C_/δ_H_ 39.5/2.49 ppm), and the other signals were normalized to the same intensity of the DMSO signals (since the same DMSO volume and initial amount of sample was used in all the cases).

The HSQC experiment used Bruker’s “hsqcetgpsisp.2” adiabatic pulse program with spectral widths from 0 to 10 ppm (5000 Hz) and from 0 to 165 ppm (20,625 Hz) for the ^1^H and ^13^C dimensions. The number of transients was 64, and 256 time increments were always recorded in the ^13^C dimension. The ^1^ *J*_CH_ used was 145 Hz. Processing used typical matched Gaussian apodization in the ^1^H dimension and squared cosine-bell apodization in the ^13^C dimension. Prior to Fourier transformation, the data matrices were zero-filled to 1024 points in the ^13^C dimension. Signals were assigned by literature comparison [32, 51, 58, 69–72].

In the aromatic region of the spectrum, the C_2_–H_2_, C_5_–H_5_ and C_6_–H_6_ correlation signals were integrated to estimate the amount of lignins and the S/G ratio. In the aliphatic oxygenated region, the signals of methoxyls, and C_β_–H_β_ (or C_α_–H_α_) correlations in the side chains of sulfonated and non-sulfonated β-*O*-4′, phenylcoumaran and resinol substructures were integrated. The intensity corrections introduced by the adiabatic pulse program permits to refer the latter integrals to the previously obtained number of lignin units.

The percentage of phenolic structures was calculated by referring the phenolic acetate signal in the HSQC 2D-NMR spectra (at 20.5/2.23 ppm) to the total number of lignin aromatic units (G + S + S′). To overcome differences in coupling constants of aliphatic and aromatic ^13^C-^1^H couples, the latter was estimated from the intensity of the methoxyl signal, taking into account the S/G ratio of the sample, and the number of methoxyls of G and S units [73].

## Additional file

**Additional file 1.** Additional figures including VP cycle, and additional kinetic, Py-GC/MS, SEC and NMR results. **Fig. S1.** VP catalytic cycle and CI, CII and resting state electronic absorption spectra. **Fig. S2.** Kinetics of CI reduction by native, acetylated and permethylated softwood and hardwood lignosulfonates: Native VP vs W164S variant. **Fig. S3.** Lignosulfonate permethylation: Py-GC/MS of softwood lignosulfonate before and after 1 h methylation with methyl iodide. **Fig. S4.** SEC profiles of softwood and hardwood nonphenolic lignosulfonates treated for 24 h with native VP and its W164S variant and controls without enzyme. **Fig. S5.** HSQC NMR spectra of acetylated softwood and hardwood lignosulfonates treated for 24 h with native VP and its W164S variant, and control without enzyme. **Fig. S6.** Kinetics of reduction of LiP CII by native and permethylated softwood and hardwood lignosulfonates. **Fig. S7.** SEC profiles of softwood and hardwood lignosulfonates treated for 24 h with native LiP and controls without enzyme. **Fig. S8.** HSQC NMR spectra of native softwood and hardwood lignosulfonates treated for 3 and 24 h with LiP-H8, and the corresponding controls without enzyme. **Fig. S9.** Difference spectra of peroxidase-treated softwood lignosulfonates minus their controls. **Fig. S10.** Difference spectra of peroxidase-treated hardwood lignosulfonates minus their controls.

## Abbreviations

CI: compound I (of peroxidase catalytic cycle)
CII: compound II (of peroxidase catalytic cycle)
DHP: dehydrogenation polymer (in vitro synthesized lignin)
DTT: dithiothreitol
EDTA: ethylenediaminetetraacetic acid
G: guaiacyl (lignin unit)
HSQC: heteronuclear single-quantum correlation
*k*_2_ and *k*_3_: first-order rate constants for CI and CII reduction, respectively
*k*_2app_ and *k*_3app_: apparent second-order rate constants for CI and CII reduction, respectively
*K*_D2_ and *K*_D3_: equilibrium dissociation constants for CI and CII reduction, respectively
*k*_obs_: pseudo-first-order rate constant
LiP: lignin peroxidase
LRET: long-range electron transfer
Mp: main peak (in SEC)
NMR: nuclear magnetic resonance
Py-GC/MS: pyrolysis-gas chromatography/mass spectrometry
S: syringyl (lignin unit)
SEC: size-exclusion chromatography
VP: versatile peroxidase
