# Supplementary material for: Role of surface tryptophan for peroxidase oxidation of nonphenolic lignin
Source: Biotechnol Biofuels. 2016 Sep 17;9:198. doi: 10.1186/s13068-016-0615-x (PMC5467052; doi:10.1186/s13068-016-0615-x)
Supplement: Supplementary file 1 — Additional file 1. Additional figures including VP cycle, and additional kinetic, Py-GC/MS, SEC and NMR results. Fig. S1. VP catalytic cycle and CI, CII and resting state electronic absorption spectra. Fig. S2. Kinetics of CI reduction by native, acetylated and permethylated softwood and hardwood lignosulfonates: Native VP vs W164S variant. Fig. S3. Lignosulfonate permethylation: Py-GC/MS of softwood lignosulfonate before and after 1 h methylation with methyl iodide. Fig. S4. SEC profiles of softwood and hardwood nonphenolic lignosulfonates treated for 24 h with native VP and its W164S variant and controls without enzyme. Fig. S5. HSQC NMR spectra of acetylated softwood and hardwood lignosulfonates treated for 24 h with native VP and its W164S variant, and control without enzyme. Fig. S6. Kinetics of reduction of LiP CII by native and permethylated softwood and hardwood lignosulfonates. Fig. S7. SEC profiles of softwood and hardwood lignosulfonates treated for 24 h with native LiP and controls without enzyme. Fig. S8. HSQC NMR spectra of native softwood and hardwood lignosulfonates treated for 3 and 24 h with LiP-H8, and the corresponding controls without enzyme. Fig. S9. Difference spectra of peroxidase-treated softwood lignosulfonates minus their controls. Fig. S10. Difference spectra of peroxidase-treated hardwood lignosulfonates minus their controls. [file 13068_2016_615_MOESM1_ESM.pdf]

ADDITIONAL FILE 1: Supplemental figures

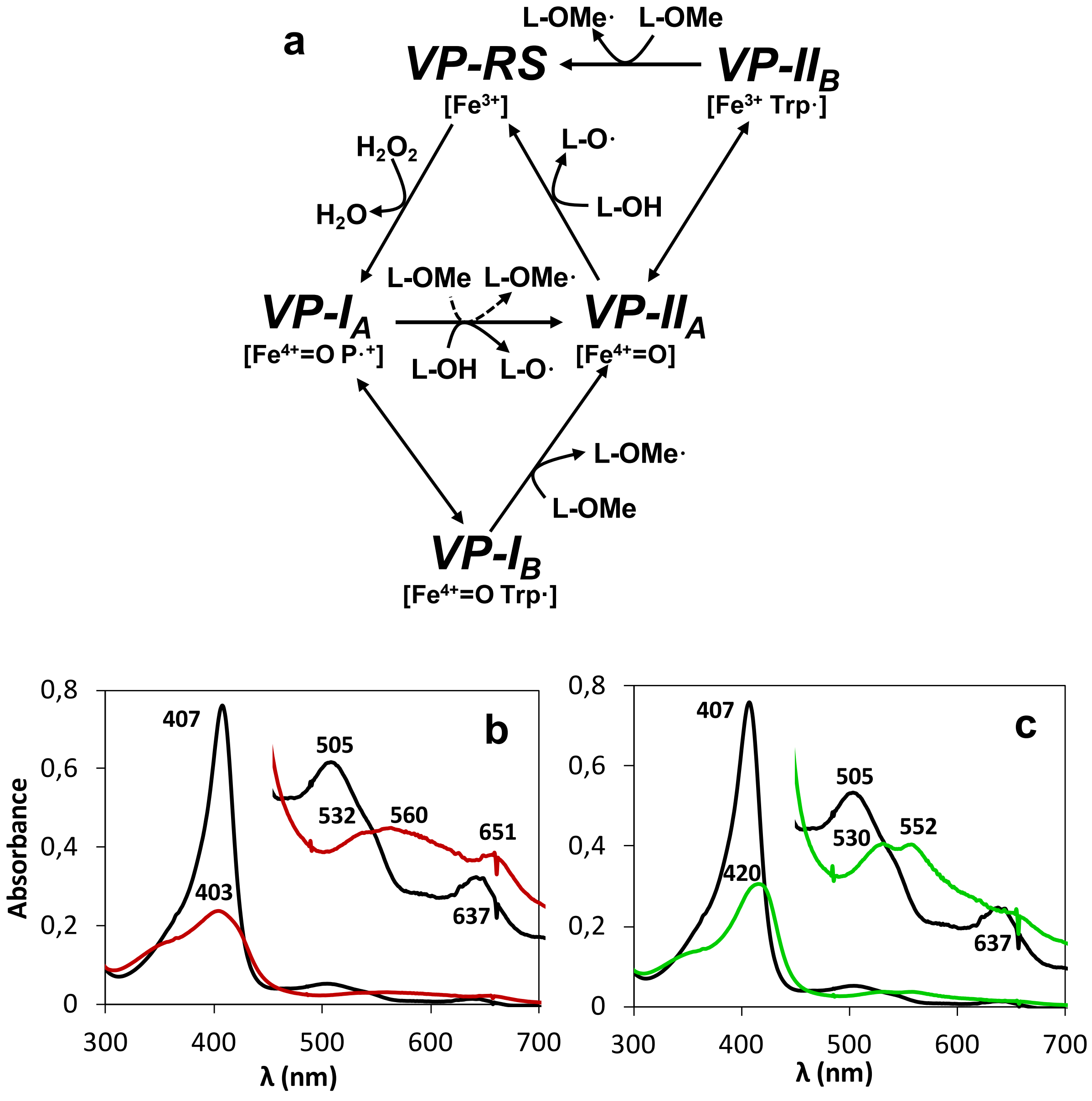

Figure S1. VP catalytic cycle and CI, CII and resting state (RS) electronic absorption spectra. **a)** Scheme for VP catalytic cycle showing RS (Fe^{3+}) activation by H_2O_2, and nonphenolic lignin (L-OMe) oxidation by a tryptophanyl radical (VP-I_B and VP-II_B) formed by one electron transfer from Trp164 to VP-I_A (Fe^{IV}=O·porphyrinyl radical, $P^{\cdot +}$, complex) and VP-II_A (Fe^{IV}=O) heme (external cycle). In contrast, phenolic lignin is directly oxidized by VP-I_A and VP-II_A (internal cycle), the former most probably being also able to oxidize nonphenolic lignin with low rates (dashed line). **b)** Spectrum of CI (red line) formed by addition of one equivalent of H_2O_2 (in 10 mM Na-tartrate pH 5) to RS (black line). **c)** Spectrum of CII (green line) after addition of two equivalents of H_2O_2 to RS (black line). Details of the 450-700 nm region in **b** and **c** are shown in x3 scale.

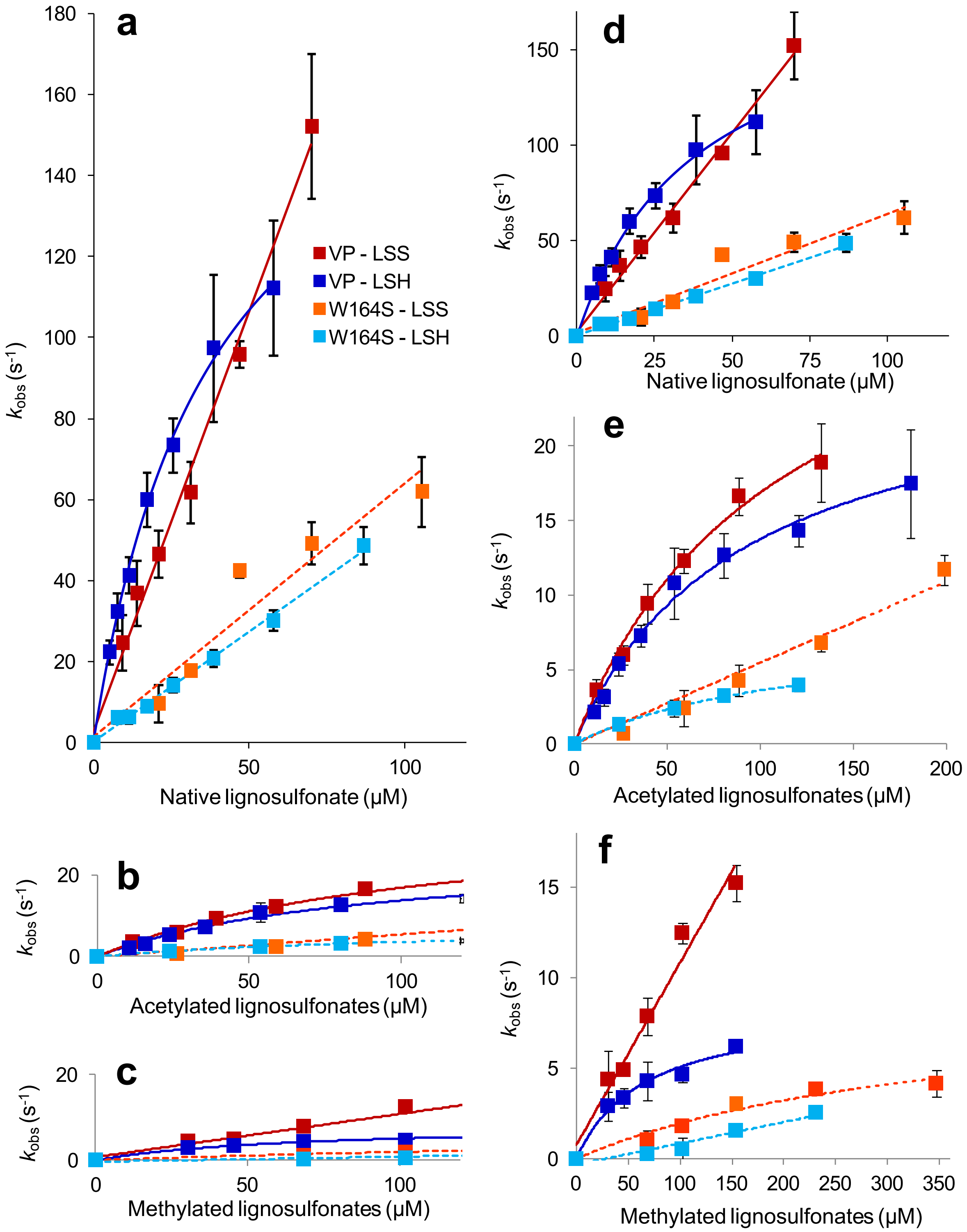

Figure S2. Kinetics of CI reduction by native (**a** and **d**), acetylated (**b** and **e**) and permethylated (**c** and **f**) softwood (LSS, red) and hardwood (LSH, blue) lignosulfonates: Native VP (continuous line) *vs* W164S variant (dashed line). In **a-c**, axes are the same for easier comparison, while in **d-f** they are expanded for better showing the different adjustments.

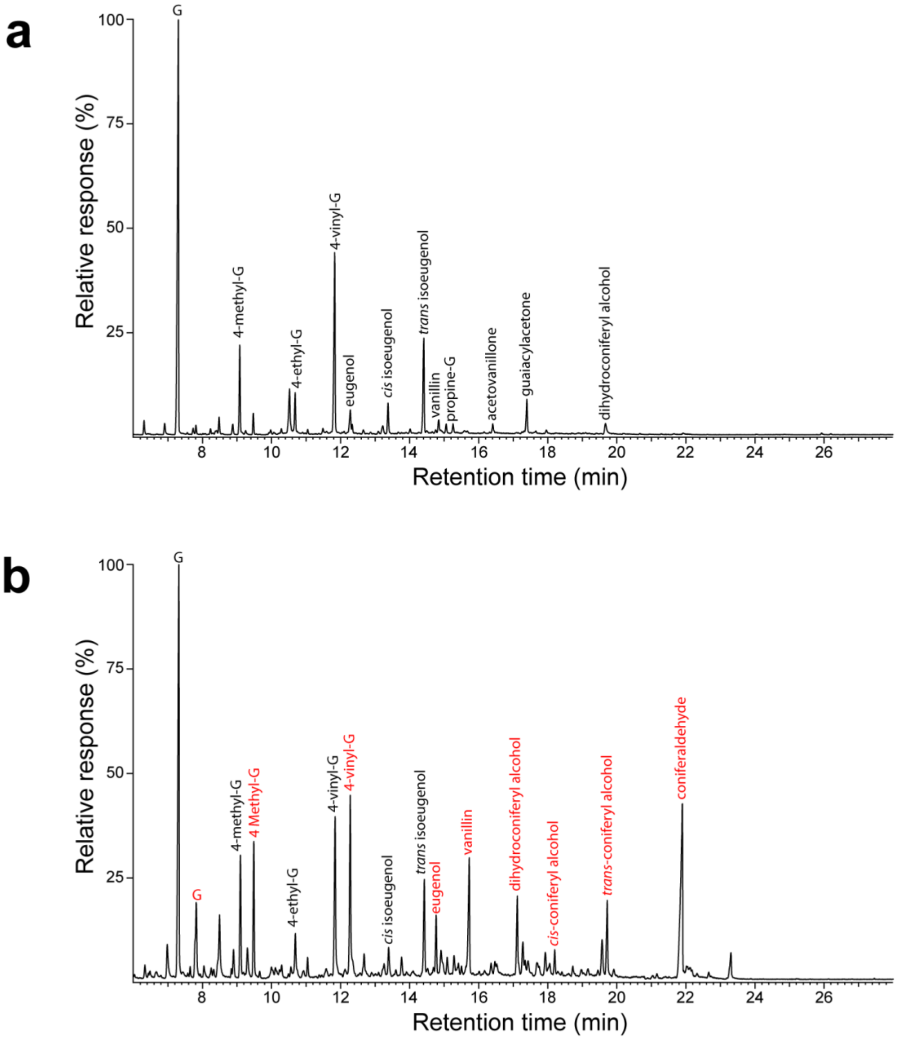

Figure S3. Pyrograms of softwood lignosulfonate before and after 1 h methylation with methyl iodide. Phenolic compounds in the native lignin pyrogram (**a**) correspond to both phenolic and nonphenolic (etherified) lignin units, while lignosulfonate methylation results in pyrogram (**b**) with both phenolic (black labels) and nonphenolic (red labels) compounds (the former originating from interunit ether breakdown in the nonphenolic moiety, and the latter from the phenolic moiety that was methylated by methyl iodide). Guaiacol (G) and 4-substituted G are among the main Py-GC/MS compounds.

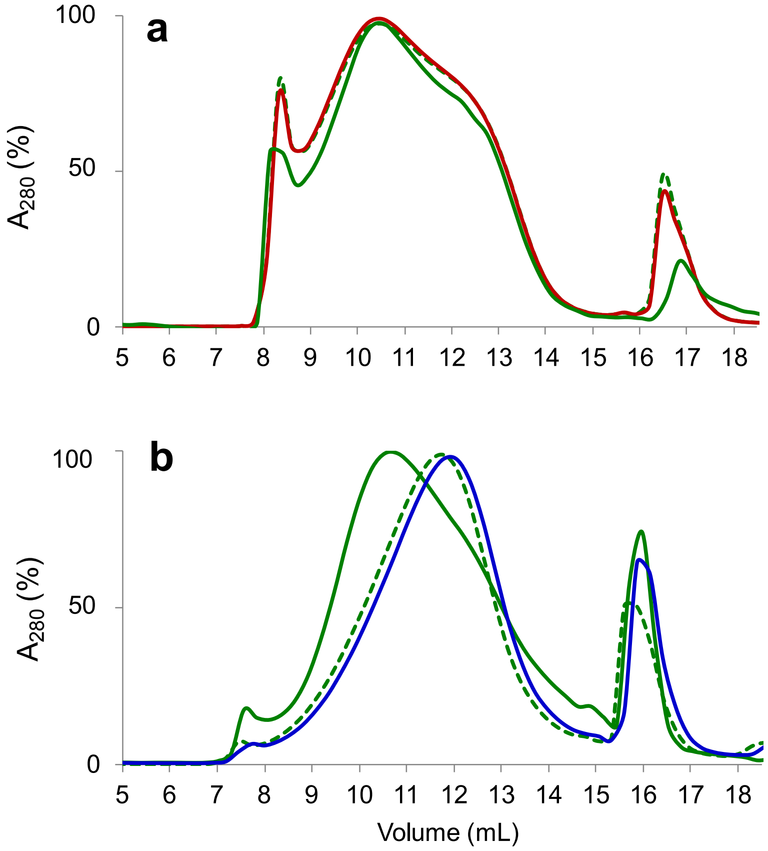

Figure S4. SEC profiles of softwood (**a**) and hardwood (**b**) nonphenolic lignosulfonates treated for 24 h with native VP (green) and its W164S variant (green dashes) and controls without enzyme (red and blue). Acetylated lignosulfonate samples (12 g·L^{-1}) after a 24-h treatment with 1.2 μM native VP and its W164S variant in presence of 9.5 mM H_2O_2, and the corresponding controls without enzyme, were analyzed in a Superdex-75 column using 0.15 M NaOH as eluent (0.5 mL·min^{-1}) and detection at 280 nm.

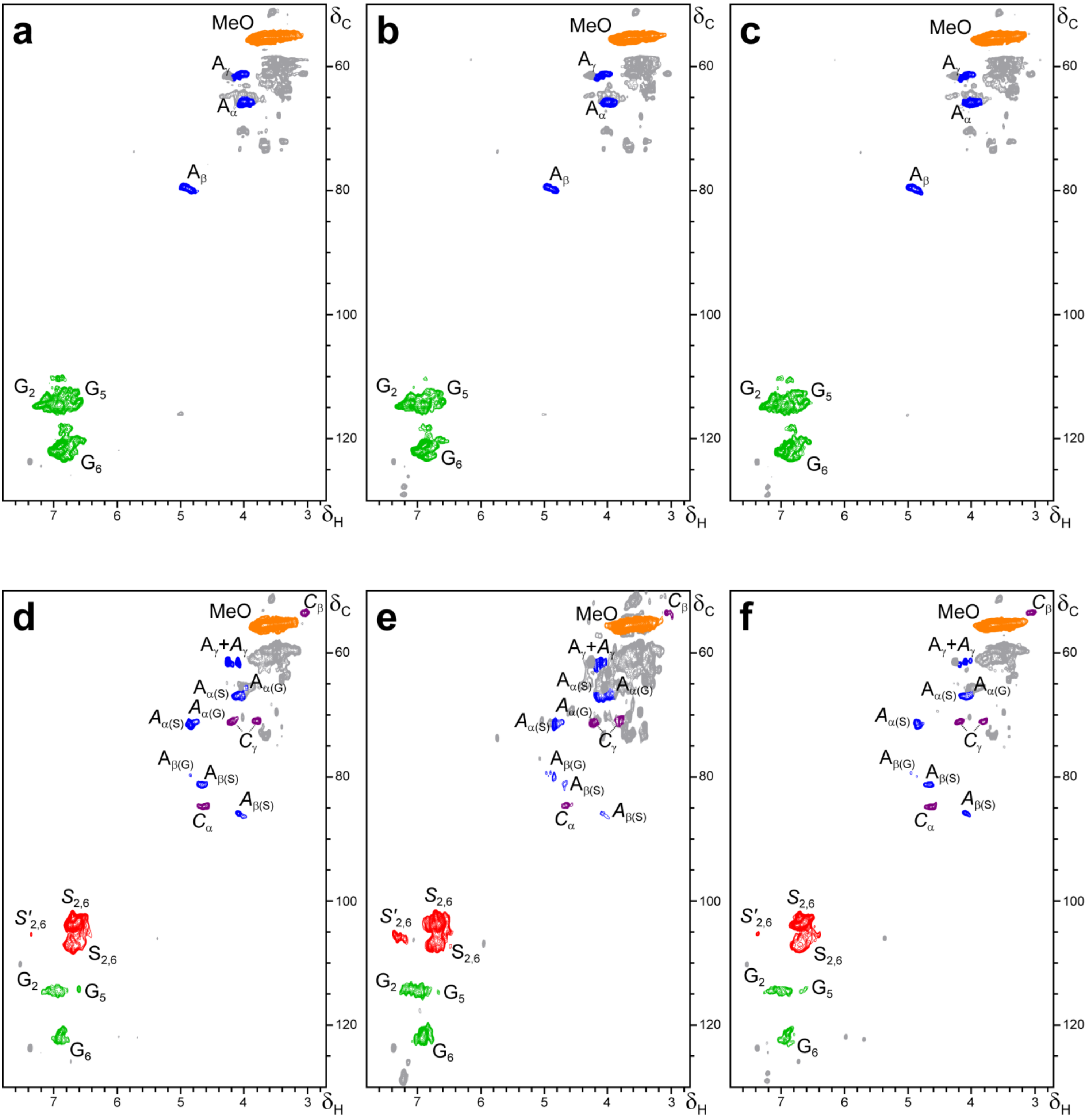

Figure S5. HSQC NMR spectra of acetylated softwood (**a-c**) and hardwood (**d-f**) lignosulfonates treated for 24 h with native VP (**b** and **e**) and its W164S variant (**c** and **f**), and control without enzyme (**a** and **d**). See **Fig. 4** for signal assignments.

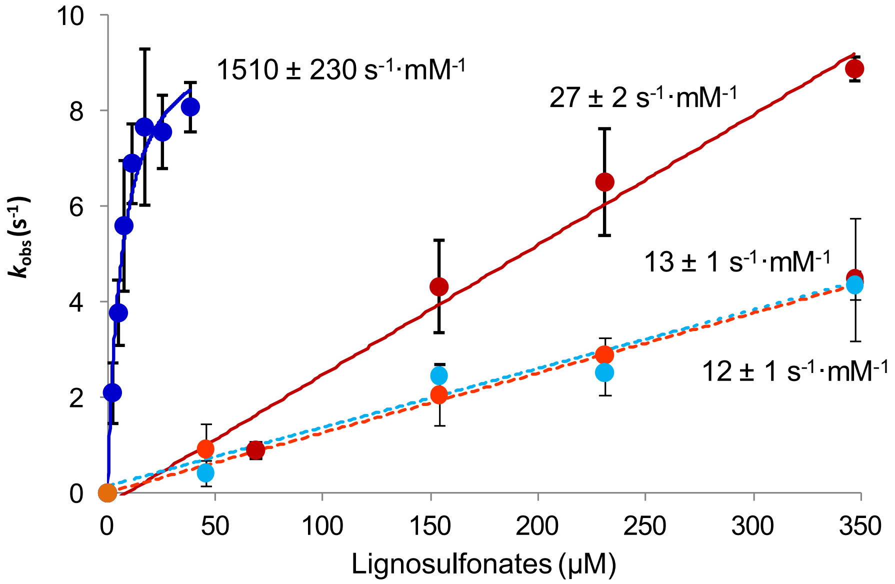

Figure S6. Kinetics of reduction of LiP CII by native (continuous lines) and permethylated (dashes) softwood (red) and hardwood (blue) lignosulfonates. The apparent second-order rate constants (k_{3app}) values are indicated. For native hardwood lignosulfonate k_3 (9.8 ± 0.6 s^{-1}) and K_{D3} (6.5 ± 1.3 μM) could be also obtained.

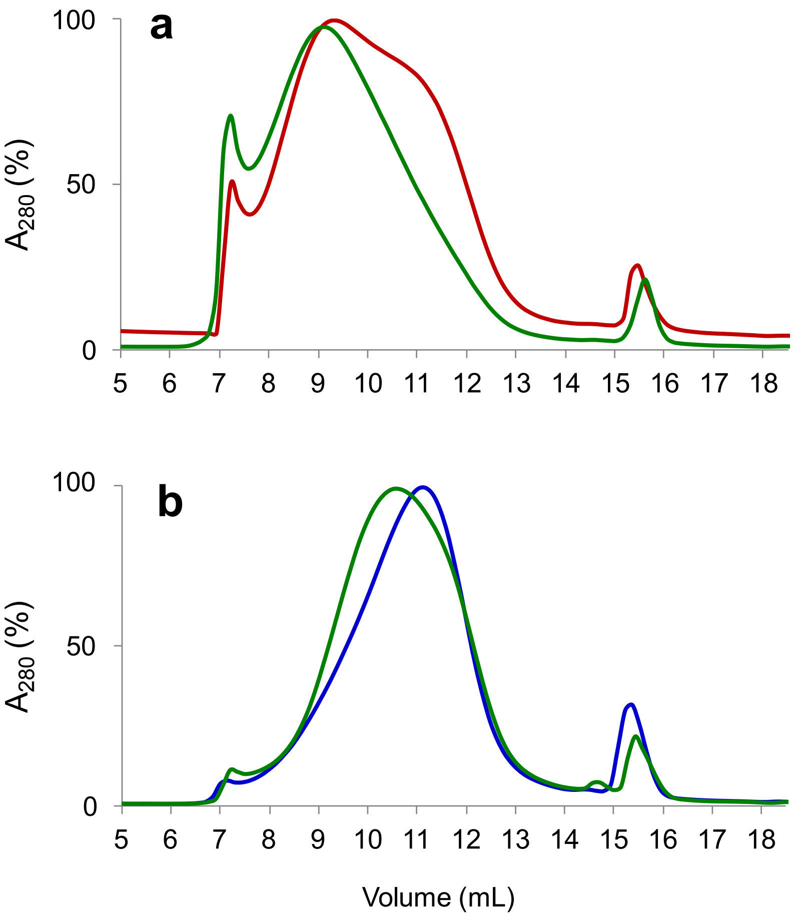

Figure S7. SEC profiles of softwood (**a**) and hardwood (**b**) lignosulfonates treated for 24 h with native LiP (green) and controls without enzyme (red and blue). Lignosulfonate samples (12 g L^{-1}) after a 24-h treatment with 1.2 μM LiP-H8 in presence of 9.5 mM H_2O_2, and the corresponding controls without enzyme, were analyzed in a Superdex-75 column using 0.15 M NaOH as eluent (0.5 mL·min^{-1}) and detection at 280 nm.

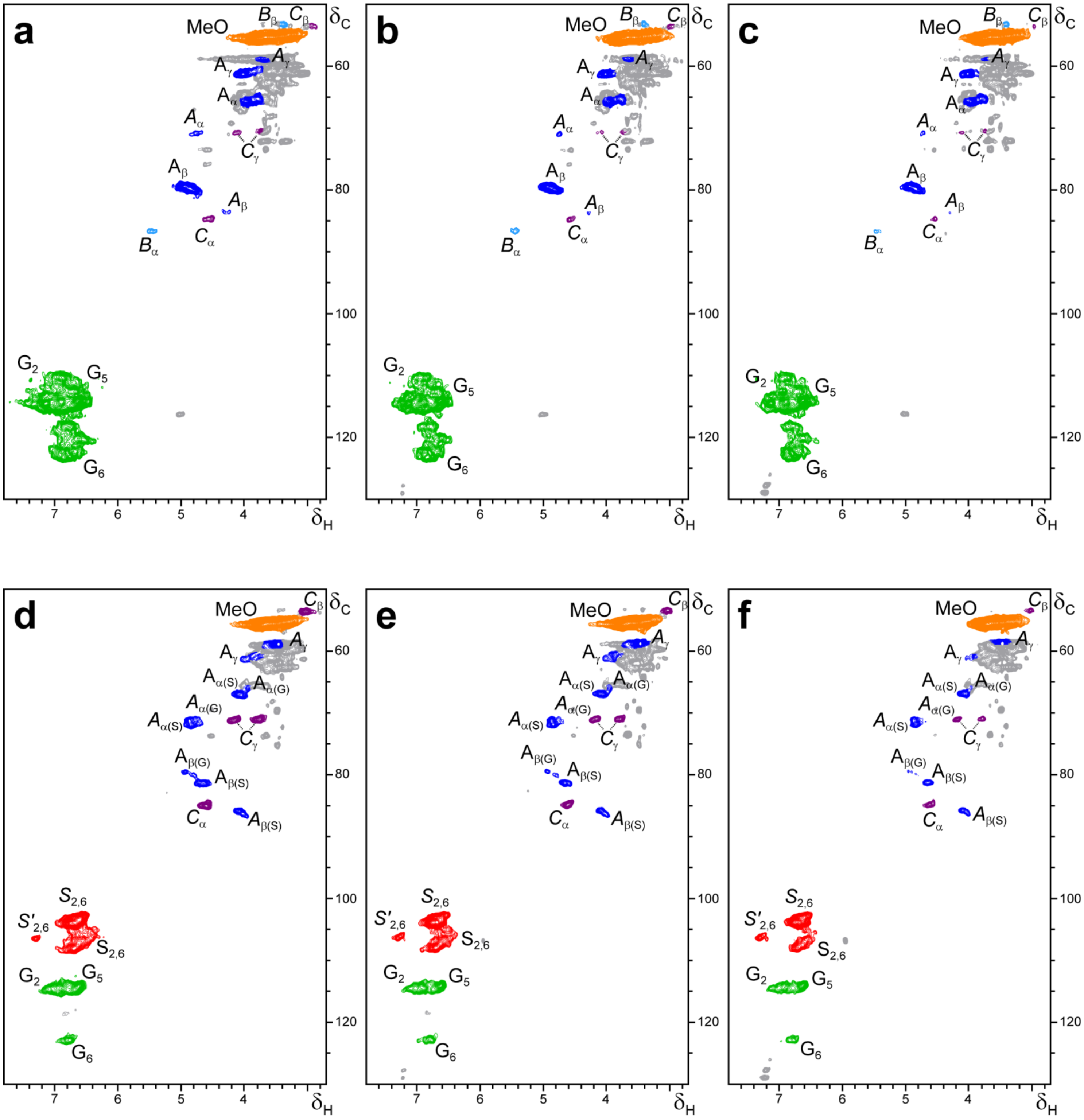

Figure S8. HSQC NMR spectra of native softwood (**a-c**) and hardwood (**d-f**) lignosulfonates treated for 3 h (**b** and **e**) and 24 h (**c** and **f**) with LiP-H8, and the corresponding controls without enzyme (**a** and **d**). See **Fig. 4** for signal assignments.

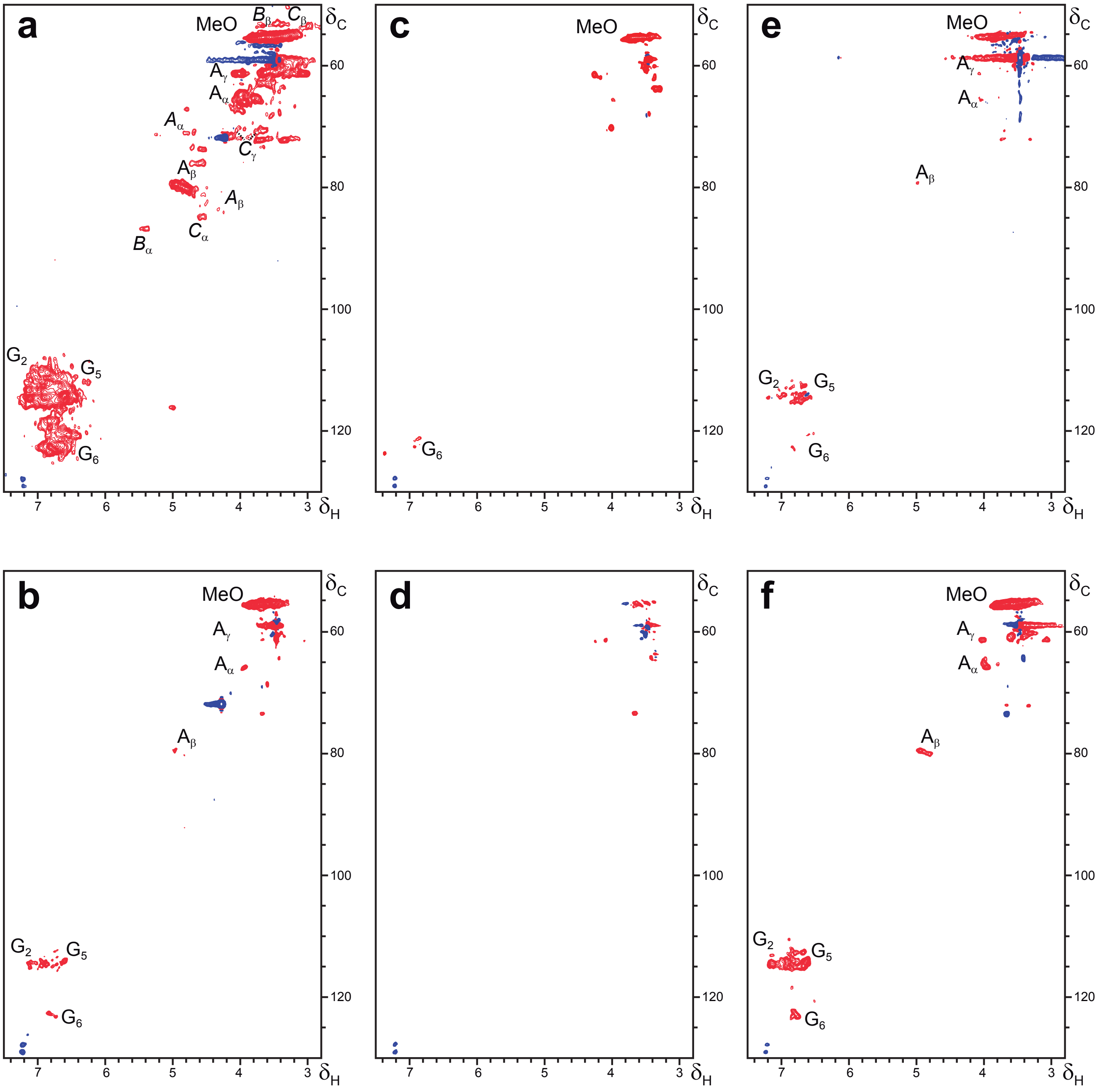

Figure S9. Difference spectra of peroxidase-treated softwood lignosulfonates minus the controls. **a)** VP treated native lignosulfonate (24 h). **b)** W164S treated native lignosulfonate (24 h). **c)** VP treated acetylated lignosulfonate (24 h). **d)** W164S treated acetylated lignosulfonate (24 h). **e)** LiP treated native lignosulfonate (3 h). **f)** LiP treated native lignosulfonate (24 h). Signals decreasing (red) and increasing (blue) intensity during treatment are shown. See **Fig. 4** for signal assignments.

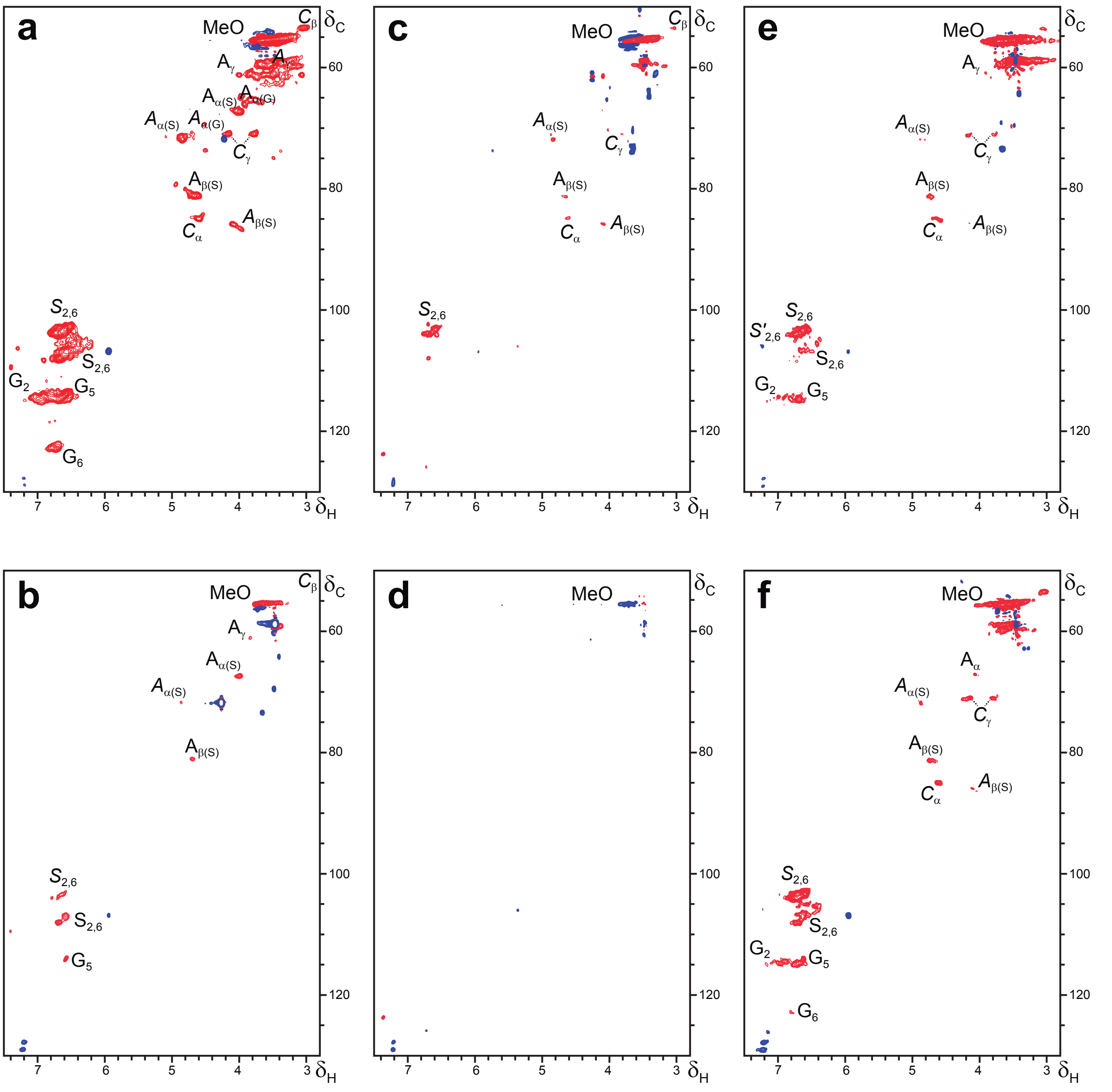

Figure S10. Difference spectra of peroxidase-treated hardwood lignosulfonates minus the controls. **a)** VP treated native lignosulfonate (24 h). **b)** W164S treated native lignosulfonate (24 h). **c)** VP treated acetylated lignosulfonate (24 h). **d)** W164S treated acetylated lignosulfonate (24 h). **e)** LiP treated native lignosulfonate (3 h). **f)** LiP treated native lignosulfonate (24 h). Signals decreasing (red) and increasing (blue) intensity during treatment are shown. See **Fig. 4** for signal assignments.
